# miRNA-22 Upregulates *Mtf1* in Dorsal Horn Neurons and Is Essential for Inflammatory Pain

**DOI:** 10.1155/2022/8622388

**Published:** 2022-02-10

**Authors:** Ling-Yun Hao, Ming Zhang, Yang Tao, Hengjun Xu, Qiaoqiao Liu, Kehui Yang, Runa Wei, Huimin Zhou, Tong Jin, Xiao-Dan Liu, Zhouya Xue, Wen Shen, Jun-Li Cao, Zhiqiang Pan

**Affiliations:** ^1^Jiangsu Province Key Laboratory of Anesthesiology, Xuzhou Medical University, Xuzhou 221004, China; ^2^Jiangsu Province Key Laboratory of Anesthesia and Analgesia Application Technology, Xuzhou Medical University, Xuzhou 221004, China; ^3^NMPA Key Laboratory for Research and Evaluation of Narcotic and Psychotropic Drugs, Xuzhou 221004, China; ^4^Department of Pain, Shanghai Tenth People's Hospital, Tongji University, Shanghai 200072, China; ^5^Department of Pain, The Affiliated Hospital of Xuzhou Medical University, Xuzhou 221002, China; ^6^Department of Anesthesiology, The Affiliated Hospital of Xuzhou Medical University, Xuzhou 221002, China

## Abstract

Chronic inflammatory pain seriously affects patients' quality of life because of a paucity of effective clinical treatments caused, at least in part, by lack of full understanding of the underlying mechanisms. miRNAs are known to be involved in inflammatory pain via silencing or degrading of target mRNA in the cytoplasm. The present study provides a novel mechanism by which miRNA-22 positively regulates metal-regulatory transcription factor 1 (*Mtf1*) in the nuclei of neurons in the dorsal horn of the spinal cord. We found that miRNA-22 was significantly increased in the dorsal horn of mice with either inflammatory pain induced by plantar injection of complete Freund's adjuvant (CFA) or neuropathic pain induced by unilateral sciatic nerve chronic constrictive injury (CCI). Knocking down or blocking miRNA-22 alleviated CFA-induced mechanical allodynia and heat hyperalgesia, whereas overexpressing miRNA-22 produced pain-like behaviors. Mechanistically, the increased miRNA-22 binds directly to the *Mtf1* promoter to recruit RNA polymerase II and elevate *Mtf1* expression. The increased *Mtf1* subsequently enhances spinal central sensitization, as evidenced by increased expression of p-ERK1/2, GFAP, and c-Fos in the dorsal horn. Our findings suggest that the miRNA-22–*Mtf1* signaling axis in the dorsal horn plays a critical role in the induction and maintenance of inflammatory pain. This signaling pathway may be a promising therapeutic target in inflammatory pain.

## 1. Introduction

Epigenetic mechanisms play a critical role in the development and maintenance of chronic pain [1–5]. microRNAs (miRNAs), a class of non-coding RNAs 20–25 nucleotides in length, are highly conserved and stable, and recent studies have shown a strong connection between miRNAs and various diseases of the nervous system. Altered miRNA expression in primary sensory neurons after nerve injury was first observed in 2010 [6]; since then, a large body of research has demonstrated the essential regulatory function of miRNAs in the initiation and progression of pain signals in primary afferent nociceptors, dorsal root ganglia (DRG), spinal cord, and various brain areas. Manipulating miRNA expression can efficiently prevent or reverse persistent inflammatory, neuropathic, and cancer pain behavior [7, 8].

Previous reports on the role of miRNAs in pain have generally focused on negative regulatory mechanisms, by which miRNAs silence or degrade mRNA when the miRNA seed region matches the 3'-UTR fragment of mRNA in the neuronal cytoplasm [9]. However, several mature miRNAs have recently been found to transfer from the cytoplasm to the nucleus, where they subsequently regulate the transcription of target genes [10]. For example, miRNA-1224, located mainly in the nuclei of spinal neurons, is downregulated by peripheral inflammation; rescuing this downregulation attenuates inflammation-induced nociceptive hypersensitivity by reducing the level of ciRNA-Filip1 in the dorsal horn [5], suggesting that nuclear miRNAs can play an important role in the pathological processes underlying inflammatory pain. Thus, understanding the role of nuclear miRNAs in pain may provide a novel approach for the management of inflammatory pain.

In previous work, we profiled miRNA expression in the dorsal horn of the spinal cord in mice and found that peripheral inflammation significantly upregulated expression of miRNA-22 [11]. Given that miRNA-22 is highly conserved across mouse and human and participates in several diseases, including Alzheimer's disease, glioma, and hepatocellular carcinoma [12–14], it is likely to be a player in the physiological and pathological processes of multiple peripheral and central nervous diseases. However, whether miRNA-22 is involved in inflammatory pain remains unknown.

In the current work, we report that miRNA-22 was enriched in neuronal nuclei in mouse dorsal horn. Upregulation of miRNA-22 in the dorsal horn was required for the development and maintenance of inflammatory pain through its positive regulation of *Mtf1* protein. Nuclear miRNA-22 in dorsal horn neurons may therefore play a critical role in the pathological processes underlying inflammatory pain.

## 2. Materials and Methods

### 2.1. Animals

Adult male Kunming mice (20-25 g) used in this study were provided by the Experimental Animal Center of Xuzhou Medical University. All animal experimental protocols were approved by the animal protection committee of Xuzhou Medical University. Every effort was made to reduce the animal's pain and the number of used animals. The animals were kept in a standard cage in a controlled environment (temperature: 23°C ±3°C, relative humidity: 25% and 45%) and light period is maintained from 6 : 00 to 18 : 00, and food and water are freely available.

### 2.2. Pain Model

Inflammatory pain model: inflammatory pain model was implemented following the method described by Larson et al. [15]. CFA (complete Freund's adjuvant) (40 *μ*l; F5881, Sigma-Aldrich, St. Louis, MO, USA) was injected into the surface of left hindpaw plantar, or the same volume of saline was used as control. Neuropathic pain model: unilateral sciatic nerve chronic constrictive injury (CCI) model was performed as described previously [5]. Mice were anesthetized with isoflurane inhalation. Skin preparation and disinfection were performed in the operating area. The sciatic nerve was exposed by blunt dissection. About 7 mm of nerve was freed adhering tissue in proximal to the sciatic trifurcation. The sciatic nerve trunk was loosely ligated with 7-0 gut, with 3 channels spaced 1 mm apart. In sham animals, sciatic nerve was exposed without ligated. The wound was sutured in layers and treated with penicillin.

### 2.3. Behavioral Test

Paw withdrawal latencies (PWL) corresponding to thermal hyperalgesia were detected by the IITC Plantar Analgesia Meter (IITC Model 336 Analgesia Meter, Series 8; IITC Life Science, USA) as described previously [16]. Double-blind detection was performed. The mice were placed in the plexiglass box on a 3 mm thick glass plate and adapted for 1 h. The movable thermal radiation device was placed under glass panels to focus onto the plantar surface of the mice left hindpaw. The time from the start of irradiation heat to elicit withdraw of the left hindpaw was defined as heat shrinkable foot the incubation period. At the beginning, the radiant heat intensity was adjusted so that the base value of the incubation period remained at 12-15 s and an automatic cut-off time is 25 s to avoid tissue damage. The left hindpaw of each animal was thermally stimulated for 6 times at interval of 5 min and the mean value was calculated.

Paw withdrawal threshold (PWT) corresponding to mechanical allodynia was detected by using von Frey filaments by up-down method. The mice were placed in the plexiglass box on a metal mesh floor and adapted for 1 h. Starting with a 0.16 g and ending with a 6.0 g filament in ascending order. The filaments vertically stimulated the plantar surface with sufficient force to cause slight bending against the paw for 5 times at 5 min interval. A brisk withdrawal or flinching of the paw was regarded as a positive response as described previously [16]. Double-blind detection was performed.

### 2.4. Spinal/DRG Tissue Collection

Mice were anesthetized with isoflurane inhalation, then the dorsal horn of lumbar spinal cord (L3-L4) was rapidly resected, and the ipsilateral and contralateral dorsal spinal horn and the DRG were separated and then put into liquid nitrogen immediately and stored at -80°C or directly used.

### 2.5. miRNA, mRNA Extraction and Reverse Transcription-Quantitative PCR (RT-qPCR)

Total RNA extraction was performed according to the instructions of Trizol (15596–026, Invitrogen, Carlsbad, CA, USA) and the quality and quantity of total RNAs were evaluated with the NanoDrop ND-2000 (Thermo Scientific, Waltham, MA, USA). RNA (0.5 *μ*g) was reverse-transcribed into single stranded cDNA using oligo (dT)_18_ (for *Mtf1*, U6 and Gapdh gene) or specific primer (5'-TTAACTGGATACGAAGGGTCCGAACACCGGTCGTATCCAGTTAAACAGTTCT-3' for miRNA-22) with reverse transcriptase M-MLV (2641, Takara, Japan). The reverse transcription of *Mtf1*, *U6 snRNA* and *Gapdh* gene was performed at 37°C for 30 min, 42°C for 30 min and 75°C for 5 min; or miRNA-22 at 16°C for 30 min and 37°C for 30 min and 75°C for 5 min. The cDNA template (1 *μ*l) was amplified by real-time quantitative PCR with the PCR primers (miRNA-22, F: 5'-TGCGGTAAGCTGCCAGTTGAAG-3', R: 5'-TACGAA GGGTCCGAACAC-3'; *Mtf1,* F: 5'-ACACCTTCGTCTGTAATCAGGA-3', R: 5'-CTGCACGTC ACACTCAAATGG-3'; *Gapdh*, F: 5'-GGTGAAGGTCGGTGTGAACG-3', R: 5'-CTCGCTCCTGGAAGATGGTG-3'; *U6 snRNA,* F: 5'-CTCGCTTCGGCAGCAC ATATACT-3'; R, 5'-ACGCT TCACGAATTTGCGTGTC-3').

Signal of each sample was determined in triplicate using SYBR Premix ExTaqII (RR820A; Takara). Reactions were carried out in a Roche Light Cycler 480 system. The expression levels of the target genes were quantified relative to *Gapdh* (an internal control of *Mtf1*) or U6 snRNA (an internal control of miRNA-22) expression (cycle threshold [Ct]) using the 2^-(*ΔΔ*CT)^ methods [17]. Any value among triplicates that had a marked difference (≥1.00) compared with the average of the other two was discarded.

### 2.6. Fluorescence in Suit Hybridization (FISH)

The experiment was performed as the described previously [11]. Briefly, anesthetized mice were subjected to sternotomy followed by intracardial perfusion with saline and 4% ice-cold paraformaldehyde phosphate buffer, and spinal cord (L3-L4) was rapidly dissected and post fixed in 4% paraformaldehyde overnight. The tissue was dehydrated in 30% sucrose solution overnight and then transversely sectioned at the thickness of 30 *μ*m. Hybridization process was carried out according to the instructions of FISH kit (Guangzhou Exon Co, China). miRNA-22 probe labeled with digoxin (5'-Dig-ACAGTTCTTCAACTGGCAGCTT-Dig-3') was hybridized in spinal section at 37°C for 16 h-72 h and subjected to wash in buffer, and was incubated in fluorescent-conjugated digoxin secondary antibody (Sangon Biotech, Shanghai, China) at 37°C for 1 h, then washed at room temperature for 10 min/time, 3 times. All solutions were prepared with nuclease-free water. Images were captured by a high-resolution digital confocal microscope (Olympus FV1000, Japan).

### 2.7. miRNA-22 Target Construction and Luciferase Reporter Assay

The identified region of *Mtf1* promoter from a mouse genomic DNA was amplified using primer pairs (F, 5'-ACGCTCGAGGGTGCGCCAGAGTCACTTAT-3' and R, 5'-AATAAGCTTTCTCAACCTGGT TCTGCACT-3'), and cloned into pGL6 plasmid (Beyotime, Shanghai, China) via XhoI and HindIII (NEB, USA) digestion. Empty pGL6 vector was used as control plasmid.

HEK293T cells were incubated in DMEM (Gibco BRL,Grand Island, NY, USA) containing 10% FBS (Gibco BRL) and 1 × 10^5^ cells per well were seeded into 24-well plates before transfection. The reporter plasmids pGL6 or pGL6-*Mtf1* were co-transfected with miRNA-22 mimics or miRNA-22 inhibitor into 293 cells using Lipofectamine 2000 (11668-027, Invitrogen). After 48 h of incubation, the cells were collected to detect luciferase activity assays using the Double luciferase reporter kit (E1910, Promega, Madison, WI, USA) in accordance with the manufacturer's instruction. The ratio of firefly luciferase activity to Renilla luciferase activity in each group (Renilla luciferase activity as an internal reference) was used as the reporter gene activity.

### 2.8. Overexpression and Knockdown Plasmid Construction

To construct miRNA-22 (Lenti-22) and *Mtf1* overexpression vector (Lenti-*Mtf1*): one insert for miRNA-22 was prepared by PCR primer pairs (F: 5'-TGCGTTTAAACATTCAAGAACCCCTCATTAGA-3', R: 5'-TTTGACGCGTCCCCAGAGGACCAGGGTT-3') or for Mtf1 by PCR primer pairs (F: 5'-TGCGTTTAAACATGGGGGAACACAGTCCAGA-3', R: 5'-TTTGACGCGTCTAGGGTGGCACGCAGG-3'); PCR products and pWPXL vector were digested by restriction endonucleases PmeI and MluI and ligated with T4 ligase. To construct miRNA-22 knockdown (LV-miRNA-22-sponge) or Mtf1 knockdown vector (LV-Mtf1-shRNA), two oligos for LV-miRNA-22-sponge (F: 5'-CGCGtACAGTTCTTCAACTGGCAGCTTgctaAC AGTTCTTCAACTGGCAGCTTgctaACAGTTCTTCAACTGGCAGCTTttttat-3', R: 5'-CGaTaaaAAAGCTGCCAAACTGAAGAACTGTtagcAAGCTGCCAAACTAAGAACTGTtagcAAGCTGCCAAACTGAAGAACTGTa-3') or two oligos for LV-Mtf1-shRNA (F: 5'-CGCGtGGAGGATGATGAAGACGATGGAACGCCATCGTCTTCATCATCCTCCTTTTTTta-3', R: 5'-CGatAAAAAAGGAGGATGATGAAGACGATGGCGTTCCATCGTCTTCATCATCCTCCa-3') were annealed and ligated to the digested PLVTHM plasmid with MluI and ClaI (NEB), respectively. All constructs were produced by the use of standard molecular methods and confirmed by DNA sequencing.

### 2.9. Lentivirus Production and Verification

The constructed core plasmid (16 *μ*g) and two envelope plasmids, PSPAX2 (12 *μ*g) and PMD2G (4.8 *μ*g), were cotransfected into HEK293T cells in a 6-well plate according to the manufacturer's instructions of Lipofectamine 2000 (11668-027, Invitrogen). The supernatant was collected at 48 h after transfection and concentrated by using a Centricon Plus-70 filter unit (UFC910096, Millipore, MA, USA). Lentivirus with titers 10^8^ TU/ml was used in the experiment. The assays of lentivirus in vitro and in vivo infection were performed according to a previous study [11]. Briefly, 20 *μ*l lentivirus and 1.5 *μ*l polybrene (1.4 *μ*g/*μ*l; H9268, Sigma-Aldrich) were added in a 24-well plate containing 1 × 10^5^ HEK293T cells and DMEM without FBS; after 24 h, the transduction medium was replaced with 500 *μ*l fresh complete medium containing 10% FBS; cells were collected at 48 h after transduction.

### 2.10. siRNA, Mimics, Inhibitor, and Lentivirus Delivery

5 *μ*l of 20 *μ*M miRNA-22 mimics, inhibitor and *Mtf1* siRNA and 1 *μ*l Lentivirus and their respective controls scrambled siRNA or an empty vector were intrathecally injected. The method of intrathecal injection was performed according to the previous study [5]. In brief, the mouse was held firmly by the pelvic girdle and then the needle of a 10 *μ*l microsyringe was punctured into L5 and L6 vertebrae of the mouse. Proper insertion of the needle into the subarachnoid space was verified by a slight flick of the tail after a sudden advancement of the needle. The sequence of mimics, inhibitor/siRNA were as follow: miRNA-22 mimics (5'-AAGCUGCCAGUUG AAGAACUGU-3'), miRNA-2 inhibitor (5'-ACAGUUCUUCAACUGGCAGCUU-3'), *Mtf1* siRNA (sense: 5'-AAGAAACAUUGAAGGUGCAACUCUUTT-3', antisense: 5'-AAGAGUUGCACC UUCAAUGUUUCUUTT-3').

### 2.11. Antibody Immunoprecipitation (ChIP)

Immunoprecipitation was carried out according to EZ CHIP Immunoprecipitation kit (17-371, Millipore) with minor modification. Briefly, spinal cord tissues were fragmented for chromatin cross-linking, followed by homogenization and ultrasound. We added 60 *μ*l protein G agarose beads in each tube to incubate with rotation for 1 h at 4°C. Then centrifuged 4000 g for 1 min at 4°C to obtain the supernatant. The supernatant was divided into two parts, in which 10 *μ*l was used as input and the remaining part (1 ml) was transferred to the new centrifuge tube. Subsequently 2 *μ*g Anti-RNA Polymerase II/IgG was added to the supernatant to incubate along with rotation overnight at 4°C. Another 60 *μ*l protein G agarose beads were added to the mixture to incubate with rotation for 1 h at 4°C and the chromatin was pulled down by magnetic beads. Finally, we digested the protein with proteinase K. Each sample was divided into two parts to undergo the extraction of RNA for miRNA-22 and DNA for *Mtf1* promoter detection.

### 2.12. RNA-DNA Immunoprecipitation

Referring the previously described [5, 18], some experimental procedures were modified. Biotin-labeled miRNA-22 probe (5'-Bio-ACAGTTCTTCAACTGGCAGCTT-3') was used to bind the *Mtf1* promoter via recognizing the target DNA regions. Spinal cord tissues were homogenized and fragmented by ultrasound. After centrifugation, 50 *μ*l of the supernatant was retained as input, and the remaining part was incubated with miRNA-22 probes or Scrambled probes (0.5 *μ*l, 50 *μ*M) overnight with rotation at 4°C. 50 *μ*l The Dyna-beads M-280 Streptavidin (11205D, Thermo Fisher Scientific, Waltham, MA, USA) was added into mixture and incubated at 4°C for 1 h. The mixture of beads-probes-DNAs was washed and NaCl (8 *μ*l, 5 M) was added into it to incubate overnight at 65°C to reverse the formaldehyde crosslinking. Finally, DNA was extracted by the use of Mag-MK Animal Genomic DNA Extraction Kit (B518721, Sangon Biotech) for *Mtf1* promoter detection.

### 2.13. Protein Extraction and Western Blot Analysis

The spinal cord tissues (L3-L4) were homogenized in a lysis buffer containing Protease Inhibitor Cocktail (p8340, Sigma-Aldrich) [19]. The protein concentration was confirmed with BCA protein concentration determination assay kit (P0010, Beyotime, Shanghai, China). The protein samples were separated by SDS-PAGE gels and transferred onto polyvinylidene fluoride membrane (IPVH00010, Millipore). The membrane was sealed with 5% skim milk for 2 h at room temperature, subsequently incubated at 4°C overnight with the primary antibodies: *Mtf1* (1 : 500; Santa Cruz Biotechnology, CA, USA), p-ERK1/2 (1 : 5000; Sigma), ERK1/2 (1 : 1000; Santa Cruz Biotechnology), GFAP (1 : 1000; Cell Signaling Technology, Danvers, MA, USA) and *β*-actin (1 : 2000; Santa Cruz Biotechnology). The membrane was washed for 5 min/time 3 times at room temperature, incubated for 2 h in the corresponding secondary antibody: HRP-labeled goat anti-mouse IgG (1 : 1000; Beyotime), HRP-labeled goat anti-rabbit IgG (1 : 1000; Beyotime) and HRP-labeled donkey anti-goat IgG (1 : 1000; Beyotime) at room temperature. The membrane was then washed for 5 min/time 6 times, the immune complexes were detected by ECL chemiluminescent assay kit (Biosharp, Guangzhou, China). Signal intensity of band analyses was conducted with ImageJ software (Alliance Q9).

### 2.14. Spinal Neuron Culture

The primary culture of spinal neurons was conducted according to the previous study [20]. The newborn mice within 24 h were directly severed heads after disinfected with 70% alcohol. The tissue fragments were digested for 45 min at 37°C with the preheated papain (20 U/ml, Sigma-Aldrich) in oxygenated divalent-free EBSS (14155063, Life Technologies, Carlsbad, CA, USA). The enzymatic digestion reaction is terminated by using 3 ml EBSS solution containing bovine serum albumin (1 mg/ml; A1933, Sigma-Aldrich), trypsin inhibitor (10 mg/ml; T6522, Sigma-Aldrich), and DNase (0.01%; D5025, Sigma-Aldrich), and mechanical separation was operated using sterile Pasteur pipettes. The homogenate was deposited on top of 4 ml of a solution of composition similar to that used above, except that the concentration of bovine serum albumin was increased to 10 mg/ml. And then the cell solution was centrifuged at 500 rpm for 5 min, and the supernatant was discarded and instead by 5 ml of culture medium. The composition of medium was as follows: MEM-*α* (Invitrogen), FCS (5% v/v; Invitrogen), heat-inactivated horse serum (5% v/v; Invitrogen), penicillin and streptomycin (50 IU/ml for each; Invitrogen), transferrin (10 mg/ml; Sigma-Aldrich), insulin (5 mg/ml; Sigma-Aldrich), putrescine (100 nM; Sigma-Aldrich), and progesterone (20 nM; Sigma-Aldrich). After trituration using sterile Pasteur pipettes, the cells were plated on 35 mm collagen-coated plastic culture dishes. Cultures were maintained in a water-saturated atmosphere (95% air, 5% CO_2_) at 37°C until use (10–15 d). Two days after the cells were seeded, cytosine arabinoside (10 *μ*M) was added to the culture medium for 24 h to inhibit glial proliferation.

### 2.15. Cellular Fraction and RNA Isolation

PARIS Kit (AM1921, Invitrogen) was used to separately isolate nuclear and cytoplasmic RNA from cultured mouse spinal neurons following the manufacturer instructions.

### 2.16. Locomotor Function

Three reflex tests were performed according to previously described procedures [5]. To test the grasping reflex, climbing tests were performed. A metal wire-mesh (0.5 mm diameter, 5 mm wide grid) was placed vertically 30 cm above the table. Each mouse started from the bottom of the mesh with its head facing downwards. After the mouse was released, the time required for it to climb all the way to the top was recorded. A maximum time of 60 s was applied for animals that could not successfully complete this task. Two sessions were performed for each mouse with a 30-min interval, and the shorter time was recorded. To test the placing reflex, we held the mouse with the hind limbs slightly lower than the forelimbs and brought the dorsal surfaces of the hindpaws into contact with the edge of a table. The experimenter recorded whether the hindpaws were placed on the table surface reflexively. To test the righting reflex, we placed the mouse on its back on a flat surface; the experimenter noted whether it immediately assumed the normal upright position. Scores for placing, grasping, and righting reflexes were based on the counts of each normal reflex exhibited in five trials.

### 2.17. Statistical Analysis

All data were presented as mean ± SEM. The data were analyzed with a one-way or two-way ANOVA or paired or unpaired Student's t test. When ANOVA was statistically significant, pairwise comparisons between means were performed by a post hoc Tukey's test. Statistical analyses of data were performed with GraphPad Prism 5. *p* <0.05 was considered statistically significant in all analyses.

## 3. Results

### 3.1. miRNA-22 Is Increased in Mouse Dorsal Horn after Nociception

**Figure 1:**
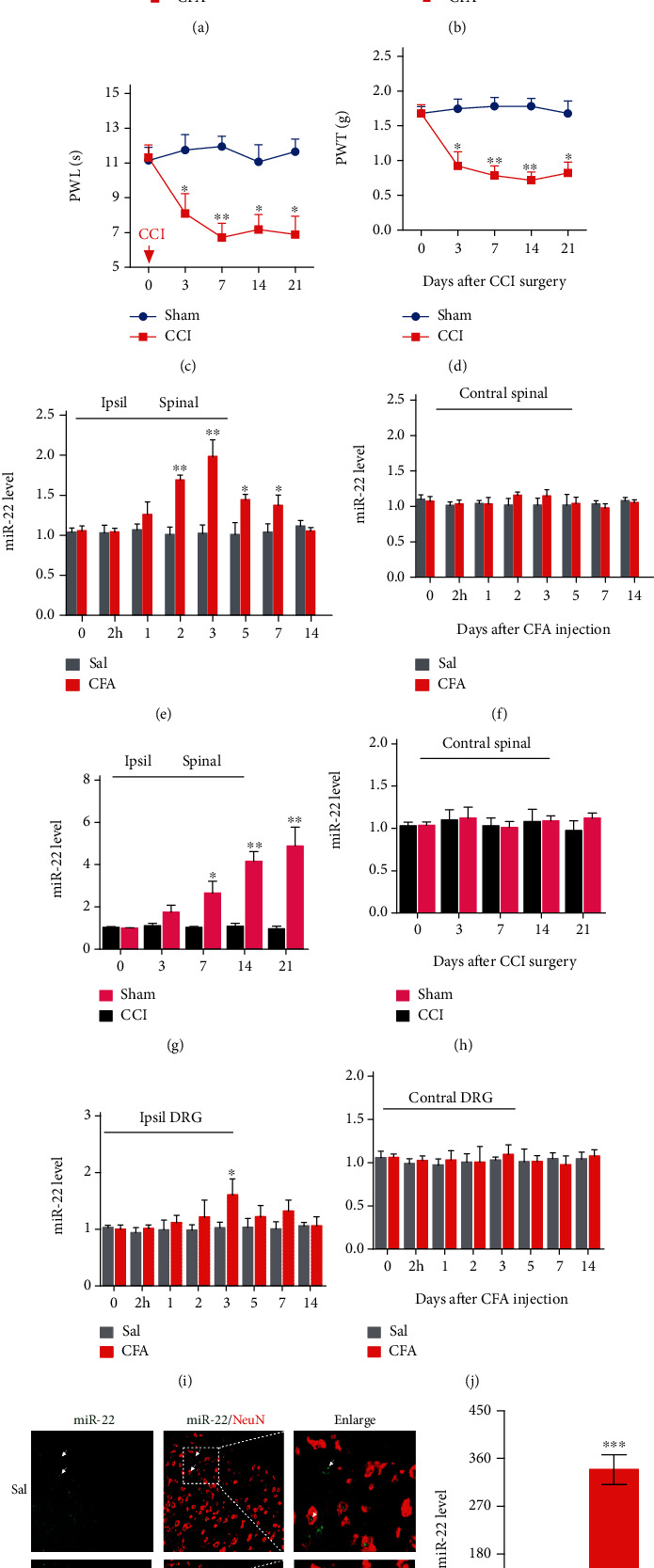
Inflammatory pain increased the expression of miRNA-22 in mouse dorsal horn. (a, b) Intraplantar injection of complete Freund's adjuvant (CFA) induced thermal hyperalgesia (a) and mechanical allodynia (b). n =6. Red arrow indicates CFA or saline (Sal) injection. 2 h represents 2 hours after CFA injection. ∗p <0.05; ∗∗p <0.01 versus Sal. Data were analyzed with a two-way repeated-measures ANOVA followed by post hoc Tukey test. (c, d) Chronic constriction injury (CCI) of unilateral sciatic nerve caused hypersensitivity to heat (c) and mechanical (d) stimuli. ∗p <0.05; ∗∗p <0.01 versus Sham. n =6. Data were analyzed with a two-way repeated-measures ANOVA followed by post hoc Tukey test. (e, f) Expression of miRNA-22 at different time points in ipsilateral (Ipsil) (e) or contralateral (Contral) (f) dorsal horn of mice with CFA-induced inflammatory pain. ∗p <0.05, ∗∗p <0.01 versus day 0. n =5. 2 h represents 2 hours after CFA injection. Data were analyzed with a two-way ANOVA followed by post hoc Tukey test. (g, h) Expression of miRNA-22 at different time points in the ipsilateral (g) or contralateral (h) dorsal horn of mice with neuropathic pain after chronic constriction injury (CCI). ∗p <0.05, ∗∗p <0.01 versus day 0. n =5. Data were analyzed with a two-way ANOVA followed by post hoc Tukey test. (i, j) Inflammatory pain slightly increased the expression of miRNA-22 in ipsilateral dorsal root ganglion (DRG) of mice (i), but not contralateral DRG (j), on only day 3 after CFA injection. ∗p <0.05 versus 0 day. n =5. Data were analyzed with a two-way ANOVA followed by post hoc Tukey test. (k) Co-staining of miRNA-22 *FISH* (green) and NeuN (a neuronal marker, red) immunofluorescence in dorsal horn on day 3 after CFA or saline injection. Scale bar, 50 *μ*m. Arrows represent the location of the miRNA-22 signal in spinal neurons and the dotted lines indicate the region enlarged in the right-hand panel. (l) Distribution of miRNA-22 in the nucleus (Nuc) and cytoplasm (Cyt) of neurons cultured *in vitro*. Neurons were cultured *in vitro* for 48 h followed by separation of cytoplasmic and nuclear RNA. ∗∗∗p <0.001 compared with Cyt group. n =4. Data were analyzed with a Student's t test.

Our previous miRNA profiling showed that peripheral inflammation caused by intraplantar injection of complete Freund's adjuvant (CFA) induced an increase in miRNA-22 in mouse dorsal horn [11]. To investigate the role of miRNA-22 in chronic pain further, we examined the time course of miRNA expression in both an inflammatory pain model (intraplantar injection of CFA) and a neuropathic pain model (chronic constriction injury (CCI) of the sciatic nerve). Consistent with previous studies [5, 21], CFA injection led to heat hyperalgesia (Figure 1(a)) and mechanical allodynia (Figure 1(b)) in mice from day 2 to day 7; similarly, peripheral nerve injury resulted in heat hyperalgesia (Figure 1(c)) and mechanical allodynia (Figure 1(d)) from day 3 to day 21 post-CCI surgery. Next, we examined the level of dorsal horn miRNA-22 at different time points after CFA injection or CCI surgery. The level of miRNA-22 in the ipsilateral dorsal horn increased by 59.78% on day 2 after CFA injection, by 87.53% on day 3, by 36.47% on day 5, and by 29.57% on day 7, but was not increased at 2 hours, on day 1, or on day 14 (Figure 1(e)); no increases were observed in the contralateral dorsal horn (Figure 1(f)). Similarly, increased levels of miRNA-22 were observed in the ipsilateral dorsal horn from day 7 to day 21 after CCI surgery (Figure 1(g)), but there were no increases in the contralateral dorsal horn (Figure 1(h)). Interestingly, CFA contributed to a transient increase in the level of miRNA-22 in the ipsilateral dorsal root ganglion (DRG) on only day 3 post-CFA injection (Figure 1(i)), with no changes in the contralateral DRG (Figure 1(j)). These results suggest that both peripheral inflammation and peripheral nerve injury cause upregulation of miRNA-22 in the ipsilateral dorsal horn. To further determine the distribution of miRNA-22 in spinal neurons, we conducted miRNA-22 fluorescence in situ hybridization (FISH) and found that miRNA-22 was co-expressed with NeuN (a neuronal marker) in dorsal spinal horn (Figure 1(k)). Furthermore, we measured the cytoplasmic and nuclear RNA from spinal neurons cultured from three-week-old mice. The subcellular analysis showed that the level of nuclear miRNA-22 was 267.15% greater than the level of cytoplasmic miRNA-22 (Figure 1(l)). These data indicate that miRNA-22 is distributed predominantly in the nucleus of spinal neurons.

### 3.2. Blocking the miRNA-22 Increase in Dorsal Horn Mitigates Inflammatory Pain

**Figure 2:**
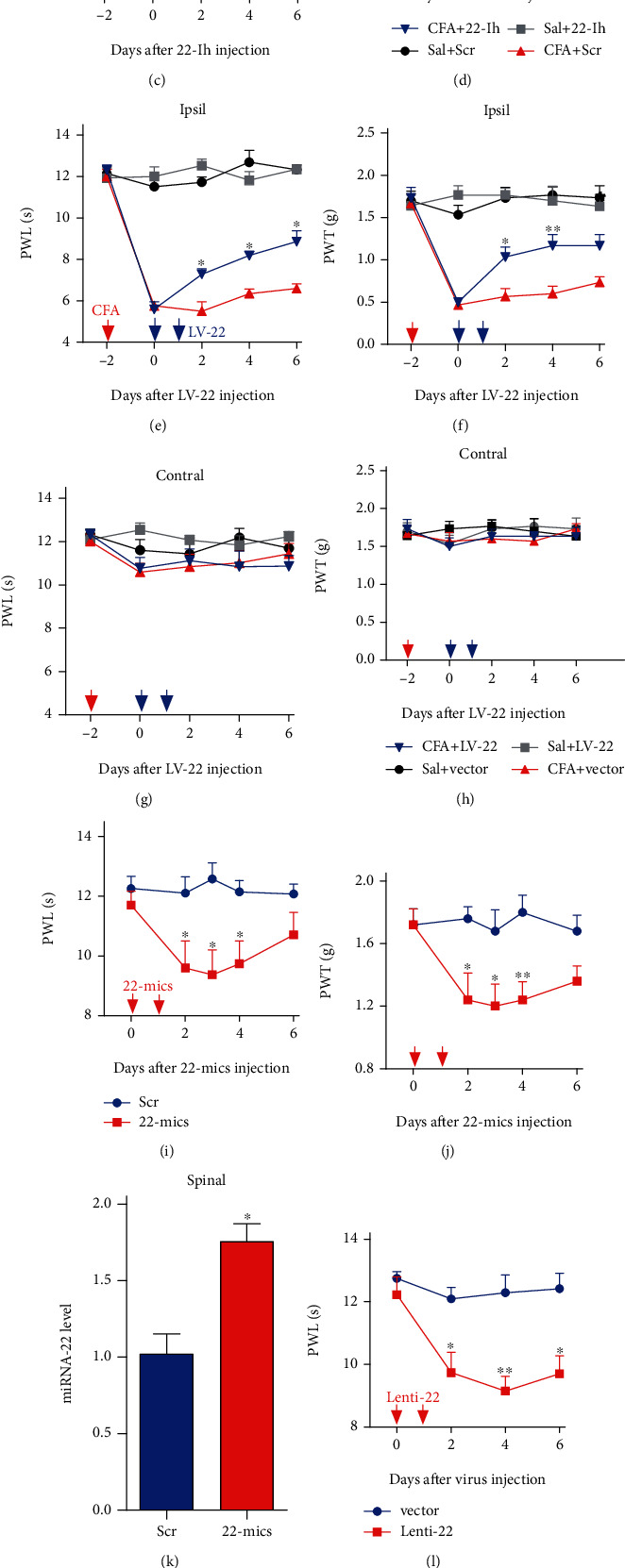
Increased miRNA-22 levels contribute to inflammatory pain behavior. (a) -(d) Intrathecal injection of miRNA-22 inhibitor for 2 consecutive days reversed CFA-induced hypersensitivity to thermal (a) and mechanical (b) stimuli on the ipsilateral paw, but did not change the sensitivity to thermal (c) and mechanical (d) stimuli in the contralateral paw in CFA-injected mice. ∗p <0.05, ∗∗p <0.01 versus CFA + Scrambled control (Scr), n =6. Red arrow represents CFA or saline injection. Blue arrows represent injection of miRNA-22 Inhibitor (22-Ih) or the scrambled control (Scr). Data were analyzed with a two-way repeated-measures ANOVA followed by post hoc Tukey test. (e)-(h) Intrathecal injection of LV-miRNA-22-sponge (LV-22) for 2 consecutive days reversed the hypersensitivity to thermal (e) and mechanical (f) stimuli in the ipsilateral paw, but did not change the sensitivity to thermal (g) or mechanical (h) stimuli in the contralateral paw in CFA mice. ∗p <0.05, ∗∗p <0.01 versus CFA + Vector. n =6. Red arrow represents CFA or saline injection. Blue arrows represent LV-22 or vector injection. Data were analyzed with a two-way repeated-measures ANOVA followed by post hoc Tukey test. (i)-(j) Intrathecal injection of an miRNA-22 mimic (22-mics) for 2 consecutive days led to the thermal (i) and mechanical (j) hypersensitivity. ∗p <0.05, ∗∗p <0.01 versus Scr. n =6. Red arrows represent 22-mics or Scr injection. Data were analyzed with a two-way repeated-measures ANOVA followed by post hoc Tukey test. (k) Intrathecal injection of 22-mics for 2 consecutive days increased the miRNA-22 level in naïve mice. n =4. ∗p <0.05, ∗∗p <0.01 versus Scr. Data were analyzed with a Student's t test. (l)-(m) miRNA-22 overexpression by intrathecal injection of Lenti-22 for 2 consecutive days increased the sensitivity to thermal (l) and mechanical (m) stimuli. ∗p <0.05, ∗∗p <0.01 versus Vector. n =6. Red arrows represent Lenti-22 or vector control injection. Data were analyzed with a two-way repeated-measures ANOVA followed by post hoc Tukey test. (n) Intrathecal injection of Lenti-22 for 2 consecutive days increased the level of miRNA-22 in naïve mice. n =6. ∗∗p <0.01 versus Vector. Data were analyzed with a Student's t test.

Next, we examined whether miRNA-22 regulates inflammatory pain (Figure 2(a)–2(h)). We used two manipulation tools to reduce the binding of miRNA-22 to its downstream genes in dorsal horn: (i) 22-Ih, a synthesized miRNA-22 inhibitor (a small RNA with reverse complementary sequence to miRNA-22) and (ii) LV-22, an LV-miRNA-22-sponge (lentivirus-expressed RNA with the ability to absorb miRNA-22). As expected, CFA-induced thermal hyperalgesia and mechanical allodynia were markedly alleviated after intrathecal injection of 22-Ih on two consecutive days, but the scrambled control (Scr) had no effect (Figure 2(a), 2(b)). The therapeutic effect lasted for 4 days for thermal hyperalgesia and 2 days for mechanical allodynia. Similarly, inhibition of miRNA-22 by intrathecal injection of LV-22, but not the vector control group, significantly ameliorated pain hypersensitivity, as indicated by an increase in paw withdrawal threshold to heat and mechanical stimulation (Figure 2(e), 2(f)). Neither 22-Ih nor LV-22 changed the basal responses on the contralateral side of CFA-injected mice (Figure 2(c), 2(d) and 2(g), 2(h)), the basal responses on the ipsilateral and contralateral sides of saline-injected mice, or the animals' locomotor functions (Table S1). Collectively, these data strongly suggest that increased miRNA-22 in the dorsal horn is required for the development and maintenance of inflammatory pain.

### 3.3. Mimicking the Increase in miRNA-22 Produces Pain Hypersensitivity

We next explored whether increased miRNA-22 in dorsal horn is sufficient for inflammatory pain. We intrathecally injected a mimic of miRNA-22 (22-mics; synthesized small RNA with the same sequence as miRNA-22) or Lenti-22 (lentivirus-expressed small RNA with the same sequence as miRNA-22) in naïve adult mice. Scrambled miRNA (Scr) and lentivirus vector (Vector) were used as the respective controls. Intrathecal injection of 22-mics induced mechanical allodynia and heat hyperalgesia, as evidenced by decreases in the paw withdrawal threshold in response to mechanical stimulation and decreases in the paw withdrawal latency in response to heat stimuli (Figure 2(i), 2(j)); these decreases occurred 1 day after injection of 22-mics. Pain hypersensitivity was not observed in the scrambled control group. As expected, a significant increase in miRNA-22 was detected 2 days after injection of 22-mics, relative to the Scr group (Figure 2(k)). Similarly, intrathecal injection of Lenti-22, but not its vector control, produced pain hypersensitivity to mechanical and heat stimulation on day 2 after injection (Figure 2(l), 2(m)), with the hypersensitivity lasting at least 5 weeks (Supplementary Figure 1). Intrathecal injection of Lenti-22 also increased the level of miRNA-22 on day 2 after injection (Figure 2(n)). None of the intrathecally injected mice displayed any locomotor impairments (Table S1). Thus, our findings indicate that miRNA-22 upregulation leads to inflammatory-pain-like symptoms.

### 3.4. miRNA-22 Positively Regulates Mtf1 Expression in Inflammatory Pain

**Figure 3:**
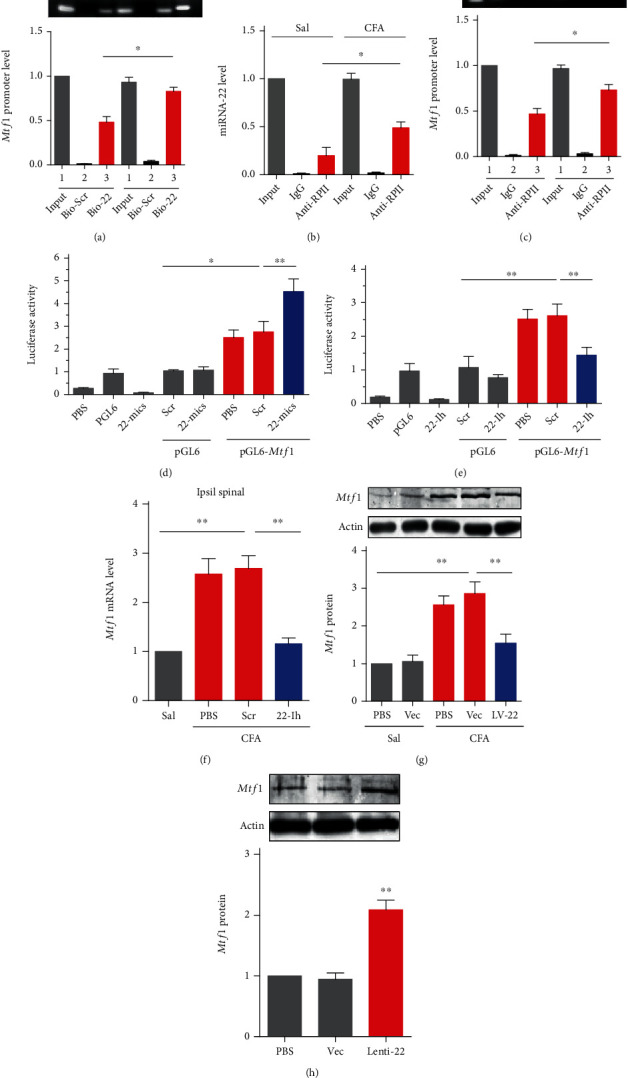
miRNA-22 positively regulates *Mtf1* expression by targeting the *Mtf1* promoter. (a) miRNA-22 binding to *Mtf1* promoter fragment evaluated by chromatin immunoprecipitation of bio-labeled miRNA-22 probes. ∗p <0.05 versus saline group. n =4. Bio-miRNA-22 probes (Bio-22) or Bio-scrambled probes (Bio-Scr) were used to pull down the *Mtf1* promoter in dorsal horn homogenate. PCR and DNA gel electrophoresis were used to test the binding accounts. Data were analyzed with a one-way ANOVA followed by post hoc Tukey test. (b) Immunoprecipitation of RNA polymerase II and miRNA-22. The complex of miRNA-22 and RNA polymerase II was pulled down with an antibody against RNA polymerase II (anti-RP II) in dorsal horn tissue collected on day 3 after CFA injection. ∗p <0.05 versus saline group. n =4. Data were analyzed with a one-way ANOVA followed by post hoc Tukey test. (c) Analysis of RNA polymerase II binding to *Mtf1* promoter in a chromatin immunoprecipitation complex by anti-RP II. ∗p <0.05 versus saline group. n =4. Data were analyzed with a one-way ANOVA followed by post hoc Tukey test. (d), (e) Luciferase reporter analysis of miRNA-22 positive regulation of the transcription of *Mtf1* with co-transfection of the reporter plasmid and an miRNA-22 mimic (22-mics) (d) or miRNA-22 inhibitor (22-Ih) (e). ∗p <0.05, ∗∗p <0.01 versus the corresponding groups. n =5. The *Mtf1* promoter bound by miRNA-22 was inserted into the luciferase promoter in the pGL6 vector. The constructed or empty pGL6 and *Mtf1* overexpression plasmid pcDNA3.1-*Mtf1* and miRNA-22 mimic or inhibitor were co-transfected into HEK293 cells and harvested 48 h after co-transfection. PBS (phosphate buffer saline) was used as the solvent control. Data were analyzed with a one-way ANOVA followed by post hoc Tukey test. (f) Intrathecal injection of miRNA-22 inhibitor weakened the increase in *Mtf1* mRNA in the spinal cord of CFA-injected mice. ∗∗p <0.01 versus the corresponding groups. n =5. Tissues were collected on day 2 after injection. Data were analyzed with a two-way ANOVA followed by post hoc Tukey test. (g) Blocking miRNA-22 with LV-22 inhibited the increase in *Mtf1* protein in the spinal cord of CFA-injected mice, as measured by western blotting. The vector (Vec) was used as a control for LV-22. ∗∗p <0.01 versus the corresponding groups. n =5. Data were analyzed with a one-way ANOVA followed by post hoc Tukey test. (h) Overexpression of miRNA-22 with Lenti-22 increased the expression of *Mtf1* protein in the dorsal horn of naïve mice, as measured by western blotting. Vec was used as a control for Lenti-22. ∗∗p <0.01 versus Vec. n =5. Data were analyzed with a one-way ANOVA followed by post hoc Tukey test.

How does the increase in miRNA-22 regulate inflammatory pain? Growing evidence suggests that miRNAs can regulate gene expression in multiple ways. For example, miRNA can silence genes by binding to the 3' un-translated region (UTR) of mRNA [10]. Additionally, miRNA can enhance gene transcription via binding to the gene promoter [22]. Because metal-regulatory transcription factor 1 (*Mtf1*) was increased in mouse spinal cord after plantar CFA injection (Figure 3(c), 3(f), 3(g)), we hypothesized that nuclear miRNA-22 in spinal neurons increases *Mtf1* expression by binding to the *Mtf1* promoter to recruit RNA polymerase II. Our informatics analysis shows that the *Mtf1* promoter contains 12 potential binding sites spanning 1157 bp from −516 ~ +640 bp region (transcription start site denoted as +1). A chromatin immunoprecipitation (ChIP) assay revealed that an *Mtf1* promoter fragment containing binding sites could be pulled down by a bio-labeled miRNA-22 probe in dorsal horn nuclear fractions from saline control mice using a PCR test and DNA agarose gel electrophoresis. This amplification did not occur with the scrambled control probe, indicating specific binding of miRNA-22 to the *Mtf1* promoter (Figure 3(a)). Furthermore, CFA injection dramatically elevated the degree of miRNA-22–promoter binding, evidenced by a 71.25% increase in band intensity in the CFA group compared with the saline group on day 3 after CFA injection (Figure 3(a)). These results suggest that chromatin in the *Mtf1* promoter region is more open when the animal is in an inflammatory pain state.

Does the increased open region bound by miRNA-22 enhance the recruitment of RNA polymerase II? As expected, in a ChIP test with anti-RNA polymerase II (Anti-RPII), CFA injection increased the binding activity of RNA polymerase II in the promoter region bound by miRNA-22: activity was elevated by 146.42% in CFA-injected mice compared with saline mice (Figure 3(b)). IgG amplification did not occur in either saline or CFA-injected mice (Figure 3(b)). Additionally, in CFA-injected mice, pull-down of the miRNA-22-bound fragment in the *Mtf1* promoter by anti-RPII was 1.5 times that of saline mice; the bound fragment was undetected in the control IgG for both groups (Figure 3(c)). To further demonstrate the role of miRNA-22 in the regulation of *Mtf1* transcription, we designed *in vitro* and *in vivo* tests. First, *in vitro*, we inserted the *Mtf1* promoter containing the regions bound by miRNA-22 upstream of the luciferase gene in the pGL6 plasmid (pGL6-*Mtf1*). We then co-transfected this reporter construct and 22-mics (the miRNA-22 mimic) in HEK293 cells, which express miRNA-22 endogenously. Reporter activity showed an increase with the empty pGL6 vector (Figure 3(d)), indicating that endogenous miRNA-22 may have activated luciferase expression. miRNA-22 overexpression (22-mics) elevated reporter activity by 64.14% compared with the pGL6-*Mtf1* plus scrambled miRNA group (Figure 3(d)). By contrast, blocking miRNA-22 with its inhibitor (22-Ih) significantly reduced reporter activity relative to the scrambled control (Figure 3(e)). These *in vitro* data indicate that miRNA-22 promotes gene expression via binding to the *Mtf1* promoter.

Next, we examined whether miRNA-22 positively regulates *Mtf1* expression *in vivo*. Blocking miRNA-22 by intrathecal injection of 22-Ih markedly inhibited the increase in *Mtf1* mRNA (Figure 3(f)) in CFA-injected mice, but the scrambled control did not. Similar results were seen with LV-22 treatment compared with the vector control (Figure 3(g)). Lenti-22-mediated overexpression of miRNA-22 increased the expression level of *Mtf1* protein (Figure 3(h)). These findings support the hypothesis that miRNA-22 participates in the increased expression of spinal *Mtf1* in CFA-induced inflammatory pain.

### 3.5. miRNA-22 Modulates Inflammatory Pain via Mediation of Mtf1

**Figure 4:**
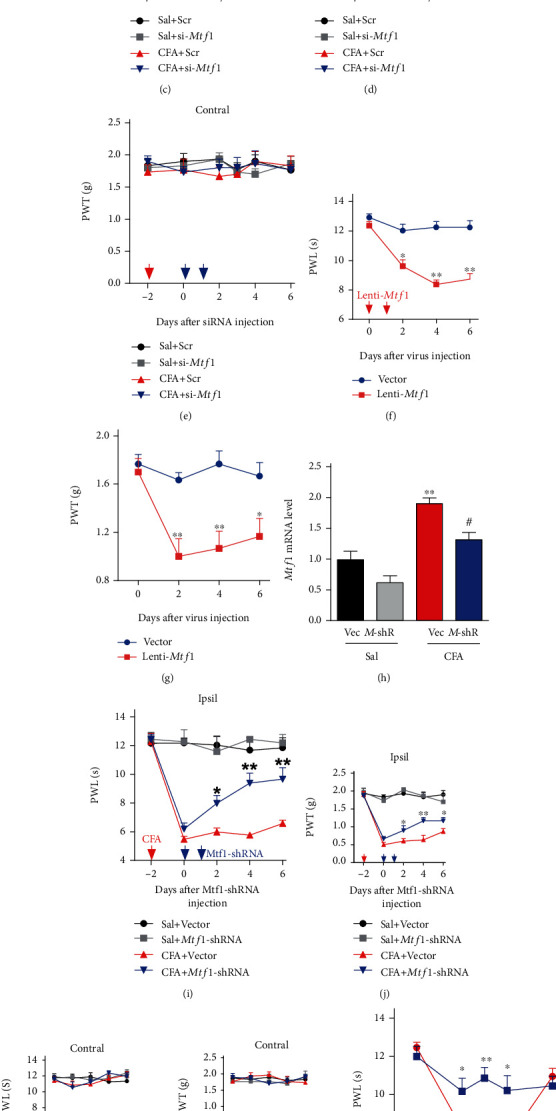
miRNA-22 mediates inflammatory pain behavior via the regulation of *Mtf1*. (a) Intrathecal injection of *Mtf1*-siRNA (si-*Mtf1*) for two consecutive days reversed the CFA-induced increase in *Mtf1* mRNA. ∗∗p <0.01 versus Sal+Scr. ^#^p <0.05 versus CFA + Scr. n =6. Data were analyzed with a two-way ANOVA followed by post hoc Tukey test. (b)-(e) Intrathecal injection of si*-Mtf1* for two consecutive days reduced thermal hyperalgesia (b) and mechanical allodynia (c) on the ipsilateral paw of CFA-injected mice, but did not affect sensitivity to thermal (d) or mechanical (e) stimuli on the contralateral paw. ∗p <0.05 versus CFA + Scr. n =6. Red arrow, CFA or saline injection. Blue arrows, si*-Mtf1* or Scr injection. Data were analyzed with a two-way repeated-measures ANOVA followed by post hoc Tukey test. (f)-(g) *Mtf1* overexpression induced by intrathecal injection of Lenti-*Mtf1* for two consecutive days induced hypersensitivity to thermal (f) and mechanical (g) stimuli. ∗p <0.05, ∗∗p <0.01 versus Vector. n =6. Red arrows indicate Lenti-*Mtf1* or the control vector injection. Data were analyzed with a two-way repeated-measures ANOVA followed by post hoc Tukey test. (h) Intrathecal injection of Lenti-*Mtf1*-shRNA (*M*-shR) for two consecutive days inhibited the increase in *Mtf1* mRNA in CFA-injected mice. ∗∗p <0.01 versus Sal+Vec. ^#^p <0.05 versus CFA + Vec. n =6. Data were analyzed with a two-way ANOVA followed by post hoc Tukey test. (i)-(l) Intrathecal injection of Lenti-*Mtf1*-shRNA (*Mtf1*-shRNA) for two consecutive days alleviated thermal hyperalgesia (i) and mechanical allodynia (j) induced by CFA injection in the ipsilateral paw, but sensitivity was unchanged in the contralateral paw (k)-(l). ∗p <0.05, ∗∗p <0.01 versus CFA + Vector. n =6. Red arrow, CFA or saline. Blue arrows, *Mtf1*-shRNA or Vector. Data were analyzed with a two-way repeated-measures ANOVA followed by post hoc Tukey test. (m)-(n) Intrathecal injection of si*-Mtf1* inhibited the pain hypersensitivity induced by miRNA-22 overexpression by its mimic (22-mics). ∗p <0.05, ∗∗p <0.01 versus 22-mics. n =6. Red arrow, 22-mics injection. Blue arrows, si*-Mtf1* injection. Data were analyzed with a two-way repeated-measures ANOVA followed by post hoc Tukey test. (o), (p) Intrathecal injection of *Mtf1*-shRNA attenuated the thermal hyperalgesia (o) and mechanical allodynia (p) induced by Lenti-22. ∗p <0.05, ∗∗p <0.01 versus Lenti-22. n =6. Red arrow, Lenti-22. Blue arrows, *Mtf1*-shRNA. Data were analyzed with a two-way repeated-measures ANOVA followed by post hoc Tukey test.

Given that increased spinal miRNA-22 is responsible for *Mtf1* upregulation during inflammatory pain, we wondered whether miRNA-22 regulates pain hypersensitivity via its effects on *Mtf1*. To test this, we first injected *Mtf1*-siRNA (si-*Mtf1*) intrathecally in the dorsal horn of CFA-injected mice to downregulate *Mtf1* expression. As expected, this manipulation (but not the scrambled siRNA control) led to a reduction in *Mtf1* expression 2 days after the intrathecal injection, and inhibited the increase in dorsal horn *Mtf1* mRNA in CFA-injected mice (Figure 4(a)). *Mtf1*-siRNA injection relieved CFA-induced pain hypersensitivity, as evidenced by a decrease in both heat hyperalgesia and mechanical allodynia from day 2 to day 4 after siRNA injection, but not the scrambled control (Figure 4(b), 4(c)). Neither *Mtf1-s*iRNA nor the scrambled control had an effect on the contralateral side in CFA-injected mice (Figure 4(d), 4(e)), or on locomotor function (Table S1). Then, we examined whether *Mtf1* upregulation by Lenti-*Mtf1* increases pain thresholds in naïve mice. Intrathecal injection of Lenti-*Mtf1,* but not the vector control, produced pain hypersensitivity to mechanical and heat stimulation on day 2 after injection (Figure 4(f), 4(g)). Next, we injected Lentivirus-*Mtf1*-shRNA (*Mtf1*-shRNA) intrathecally to knockdown *Mtf1*. As expected, we observed a decrease in *Mtf1* mRNA on day 4 post injection of *Mtf1*-shRNA, but not the vector control, in saline-injected mice, and the increase in *Mtf1* mRNA in CFA-injected mice was significantly inhibited by *Mtf1*-shRNA (Figure 4(h)). Additionally, when *Mtf1*-shRNA was injected for two consecutive days, it alleviated pain hypersensitivity (Figure 4(i), 4(j)). Neither *Mtf1*-shRNA nor the vector control had an effect on the contralateral side in CFA-injected mice (Figure 4(k), 4(l)), or on locomotor functions (Table S1).

Next, we tested whether blocking the *Mtf1* increase could ameliorate the pain hypersensitivity caused by miRNA-22 overexpression in naïve mice. Consistent with the above observations, marked pain hypersensitivity was induced by intrathecal injection of an miRNA-22 mimic on two consecutive days; these pain-like behaviors were rescued on day 1 after intrathecal injection of *Mtf1*-siNRA, but not its control, and the analgesic effect lasted for 3 days (Figure 4(m), 4(n)). Similarly, intrathecal injection of Lenti-22 over two consecutive days to upregulate dorsal horn miRNA-22 in naïve mice (Figure 2(n)) produced heat hyperalgesia and mechanical allodynia, and these pain hypersensitivities were inhibited by *Mtf1* knockdown with *Mtf1*-shRNA (injections on two consecutive days); this analgesic effect persisted for 8 days (Figure 4(o), 4(p)). Moreover, these treatments did not influence the normal locomotor activity of the mice (Table S1). Collectively, these results suggest that an increase in miRNA-22 leads to thermal hyperalgesia and mechanical allodynia via its effects on *Mtf1*.

### 3.6. miRNA-22 Regulates Central Sensitization in the Spinal Cord by Targeting Mtf1

**Figure 5:**
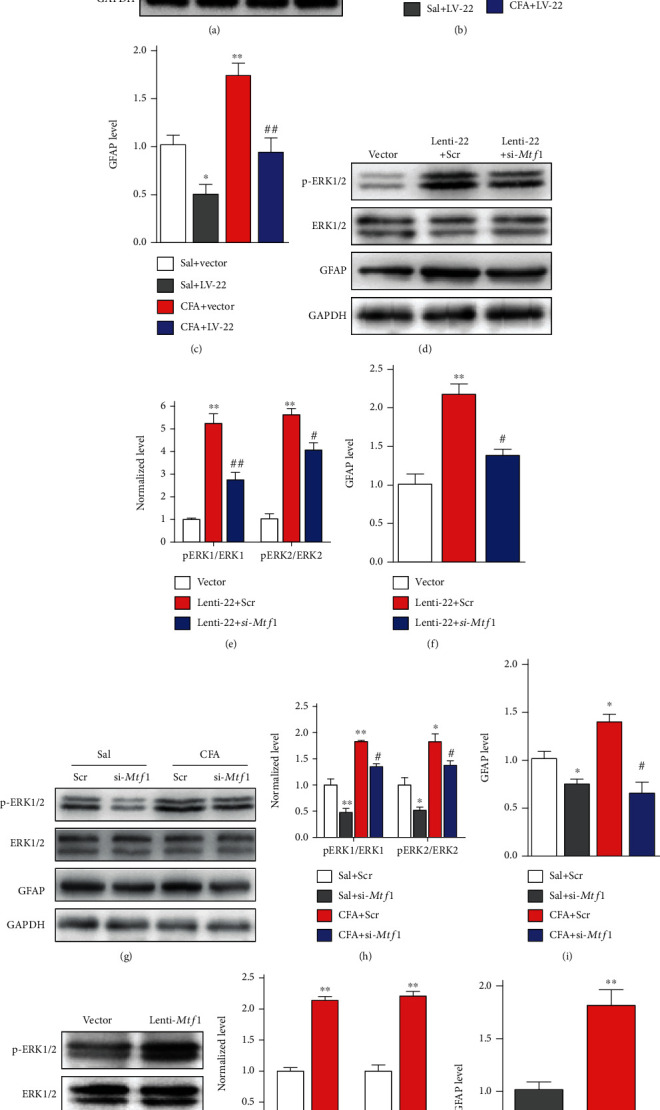
miRNA-22 activates ERK1/2 via regulation of *Mtf1*. (a)-(c) Knockdown of miRNA-22 with LV-22 reversed the CFA-induced increase in p-ERK1/2 (a, b) and GFAP protein (c) in the dorsal horn. ∗p <0.05, ∗∗p <0.01 versus Saline+Vector. n =5. ^##^p <0.01 versus CFA + Vector. n =5. Data were analyzed with a two-way ANOVA followed by post hoc Tukey test. (d)-(f) miRNA-22 overexpression induced by Lenti-22 enhanced the protein levels of p-ERK1/2 (d, e) and GFAP (f) in naïve mice, and this increase was inhibited by *Mtf1* knockdown with si-*Mtf1*. Tissues were harvested on day 2 after si-*Mtf1* following intrathecal injection of Lenti-22. ∗∗p <0.01 versus Vector. ^#^p <0.05, ^##^p <0.01 versus Lenti-22 + Scr. n =5. Data were analyzed with a one-way ANOVA followed by post hoc Tukey test. (g)-(i) siRNA-induced *Mtf1* knockdown weakened the CFA-induced increase in p-ERK1/2 (g, h) and GFAP (i) protein. ∗p <0.05, ∗∗p <0.01 versus Sal+Scr. n =5. ^#^p <0.05 versus CFA + Scr. n =5. Data were analyzed with a two-way ANOVA followed by post hoc Tukey test. (j)-(l) Intrathecal injection of Lenti-*Mtf1* increased the expression of p-ERK1/2 (j, k) and GFAP (l) protein in naïve mice. ∗∗p <0.01 versus Vector. n =5. Data were analyzed with a Student's t test.

We also tested whether increased miRNA-22 in the dorsal horn is associated with central sensitization under inflammatory pain conditions. Changes in the levels of phosphorylated extracellular signal-regulated kinase 1/2 (p-ERK1/2, a marker of neuronal activation) and glial fibrillary acidic protein (GFAP, a marker of astrocyte activation) were evaluated in the ipsilateral dorsal horn of mice subjected to manipulation by our miRNA-22 or *Mtf1* tools. We found that the levels of p-ERK1/2 and GFAP were significantly increased in the ipsilateral dorsal horn on day 3 after CFA injection and that these increases were reversed by LV-22 injections once daily for 2 days starting on day 3 after CFA injection. However, unlike p-ERK1/2, the level of total ERK1/2 did not vary among the treated groups (Figure 5(a)–5(c)). By contrast, miRNA-22 overexpression with Lenti-22, but not the control vector, augmented the levels of p-ERK1/2 and GFAP in the dorsal horn on day 4 after intrathecal injection once daily for 2 days (Figure 5(d)–5(f), Vector and Lenti-22 group), indicating that miRNA-22 positively regulates spinal sensitization. As *Mtf1* is positively involved in pain behavior, we further examined whether *Mtf1* could regulate the levels of spinal p-ERK1/2 and GFAP. *Mtf1* knockdown with siRNA, but not the scrambled control, inhibited the CFA-induced increases in p-ERK1/2 and GFAP (Figure 5(g)–5(i)) and upregulation of *Mtf1* increased the levels of p-ERK1/2 and GFAP in naïve mice (Figure 5(j)–5(l)). Furthermore, to determine whether *Mtf1* mediates the changes in sensitization produced by miRNA-22, we injected Lenti-22 intrathecally prior to the intrathecal injection of *Mtf1* siRNA. As anticipated, *Mtf1* siRNA, but not scrambled siRNA, blocked the increases in p-ERK1/2 and GFAP caused by Lenti-22. Consistent with the results above, there were no detectable changes in the level of total ERK1/2 protein with miRNA-22 overexpression (Figure 5(d)–5(f), Lenti-22 and Lenti-22 plus si-*Mtf1* groups).

**Figure 6:**
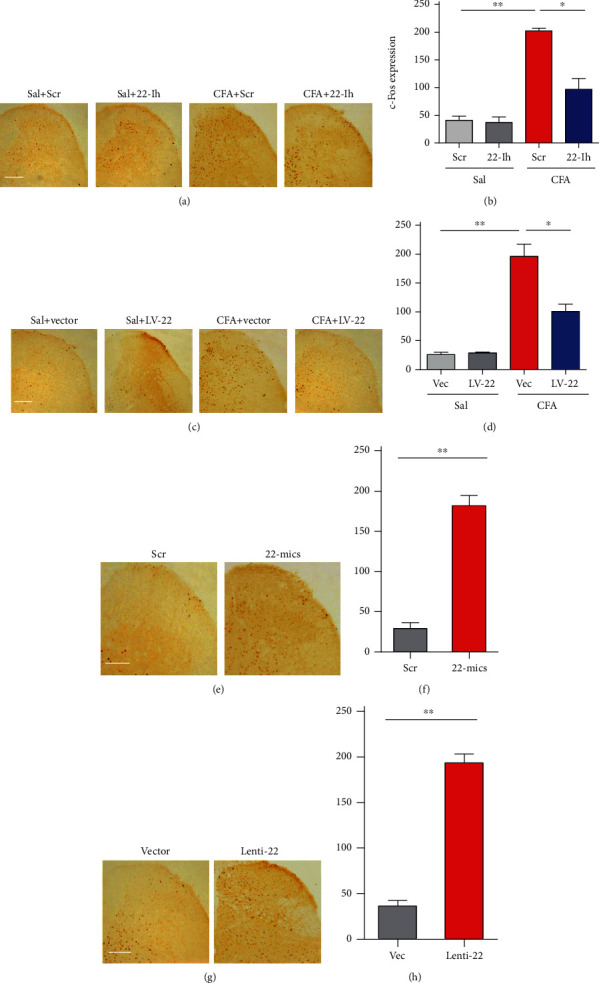
Spinal c-Fos is activated by the increase in miRNA-22 during inflammatory pain. (a)-(b) Blocking miRNA-22 with its inhibitor (22-Ih) reduced the CFA-induced increase in c-Fos expression in the ipsilateral dorsal horn. ∗p <0.05, ∗∗p <0.01 versus the corresponding groups. n =5. Data were analyzed with a two-way ANOVA followed by post hoc Tukey test. (c)-(d) Inhibiting miRNA-22 by LV-22 reduced the CFA-induced increase in c-Fos expression in the ipsilateral dorsal horn. ∗p <0.05, ∗∗p <0.01 versus the corresponding groups. n =5. Data were analyzed with a two-way ANOVA followed by post hoc Tukey test. (e)-(h) Overexpression of miRNA-22 by its mimic (22-mics, (e)-(f)) or Lenti-22 ((g)-(h)) augmented the level of c-Fos expression in the dorsal horn of naïve mice. ∗∗p <0.01 versus the corresponding groups. n =5. Scale bar, 50 *μ*m. Data were analyzed with a Student's t test.

Central sensitization is characterized by an increase in the activity of excitatory neurons during the pathogenesis of chronic pain. Therefore, we measured the level of c-Fos (a widely used marker of neuronal activity) in the dorsal horn. Immunohistochemical staining showed that downregulation of miRNA-22 by an inhibitor (22-Ih; Figure 6(a), 6(b)) or lentivirus (LV-22; Figure 6(c), 6(d)) reduced the CFA-induced increase in c-Fos expression in the dorsal horn, compared with the scrambled and vector controls. Upregulation of miRNA-22 by a mimic (22-mics; Figure 6(e), 6(f)) or Lenti-22 (Figure 6(g), 6(h)) led to a significant elevation of c-Fos expression in naïve mice. Together, these findings support the behavioral observations described above—miRNA-22 contributes to spinal sensitization through its effects on *Mtf1*.

## 4. Discussion

Chronic inflammatory pain is a complex, multi-dimensional disease. The pathogenesis of chronic inflammatory pain involves peripheral and/or central sensitization and activation of glial cells: these changes are attributed to aberrant neurotransmitter release and activity in intracellular signaling pathways at various levels in the nervous system, including the DRG, spinal cord, and brain [23–27]. The dorsal horn of the spinal cord links the peripheral nervous system to the central nervous system and is thus is a critical component in the transmission of nociception information; as such, it is a key target in the development of analgesic therapeutics and diagnostic strategies for pain. Recent reports have established a strong connection between epigenetic modifications and pain-related gene dysfunction in spinal cord neurons [3, 5, 11, 16, 28]. In this study, we provide the first evidence, to our knowledge, that peripheral inflammation leads to an increase in miRNA-22 expression in neuronal nuclei in the dorsal horn of the spinal cord. This increase is positively correlated with enhanced recruitment of RNA polymerase II to the *Mtf1* promoter and elevation of *Mtf1* mRNA and protein in spinal cord, resulting in inflammatory pain symptoms. Blocking this increase reverses the increase in RNA polymerase II recruitment, destabilizes the inflammation-induced upregulation of *Mtf1* in the spinal cord, and alleviates inflammation-associated pain hypersensitivities. Thus, our work demonstrates that miRNA-22 likely contributes to the mechanisms underlying inflammatory pain through its enhancement of *Mtf1* expression.

miRNAs are noncoding RNA species and have been well-studied in terms of their biogenesis and functions. Experiments with a variety of different pain models have linked miRNAs to multiple components at different stages within the nociceptive pathways, including ion channels [3, 29], membrane receptors [30, 31], transcription factors [32], translation factors [33], and other intracellular signals [34], in primary afferent nociceptors, DRGs, spinal cord, and brain areas. Nuclear miRNAs are known to have important functions in mRNA splicing and transcription [35, 36]. Our recent study uncovered the rich distribution of miRNA-1224 in the nuclei of spinal neurons and showed that mature circRNA-Filip1l expression is negatively regulated by miRNA-1224 via binding and splicing of the precursor to circRNA-Filip1l (pre-circRNA-Filip1l) in the spinal cord of mice with inflammatory pain. Moreover, we found that Ago2 is involved in the regulation of physiological and pathological nociception via miRNA-1224-dependent cleavage of circRNA-Filip1l [5]. However, the study of pain-related nuclear miRNAs is still in its infancy and there is currently limited molecular and functional data to support the potential regulatory role of nuclear miRNA in chronic pain. Our current work describes a novel mechanism by which miRNA-22 positively regulates *Mtf1* expression by promoting the transcription of *Mtf1* in the nucleus of spinal cord neurons.

miRNA-22 is highly conserved from fruit flies to humans and is broadly expressed in various tissues, including nervous system tissue [37]. Furthermore, miRNA-22 is associated with diabesity, glioma and hepatocellular carcinoma [13, 14, 38], dyslipidemia [39], muscle lipid metabolism [40], cardiac hypertrophy [41], rheumatoid arthritis [42], and HIV infection [43]. Recent findings show that hippocampal miRNA-22 is involved in epileptogenic focus formation [44], striatal and cortical miRNA-22 are involved in Huntington's disease [37], and peripheral blood miRNA-22 is decreased in Alzheimer's patients [12]. Although cytoplasmic miRNA-22 is closely linked to these various diseases, until now, little attention has been paid to: [1] the functional role of miRNA-22 in pain and [2] the neuronal nucleus as a locus of mechanism for miRNA-22 in the regulation of pain. In this work, we provide the first evidence that miRNA-22 is enriched in the nuclei of mouse spinal neurons. Furthermore, we revealed a novel role for miRNA-22 in the inflammatory pain process through its positive regulation of *Mtf1* in an RNA polymerase II-dependent manner. Since nuclear miRNA was first identified in HeLa cells, the existence of nuclear miRNAs has been confirmed and hundreds of nucleus-enriched miRNAs have been identified in a variety of cells [45]. Interestingly, the nuclear: cytoplasmic ratio varies across different cell lines [46]. In the present study, we found that miRNA-22 displayed the nuclear enrihment in the dorsal horn of the spinal cord, and was upregulated in the dorsal horn in mice with inflammatory or neuropathic pain, indicating that nuclear miRNA-22 in spinal neurons is closely associated with both pain models. Blocking spinal miRNA-22 markedly attenuated inflammation-induced pain behavior, and mimicking the increase in miRNA-22 induced pain hypersensitivity. However, mouse locomotor behavior was not affected by these manipulations. Our data support the hypothesis that spinal miRNA-22 contributes to the development and maintenance of inflammatory pain, but is not involved in motor function. We observed only a slight increase in miRNA-22 in the ipsilateral DRG on day 3 after CFA injection and thus excluded the possibility that DRG miRNA-22 regulates inflammatory pain. However, nociceptive information is transmitted from small DRG neurons to superficial dorsal horn neurons. Therefore, future studies should investigate whether and how miRNA-22 is involved in the transmission of nocicieptive signals between neurons in the DRG and the dorsal horn. It also remains to be seen whether miRNA-22 is enriched in the nuclei of DRG cells. In addition, the mechanism that transports miRNA-22 between the cytoplasm and the nucleus remains elusive.

In a non-classic pathway, miRNA enhances transcription through its interactions with gene promoters to recruit transcription factors or RNA polymerase [47, 48]. Because miRNA-22 is enriched in the nucleus, we speculate that miRNA-22, similarly to protein transcription factors, may regulate gene transcription through binding to the gene promoter (after unraveling of double DNA to single DNA) to enhance recruitment of RNA polymerase. Additionally, *Mtf1* was increased in the spinal cord of mice with inflammatory pain, and knockdown of spinal miRNA-22 blocked the increase in *Mtf1* mRNA and protein expression. Therefore, we wondered whether miRNA-22 regulates *Mtf1* via binding to the *Mtf1* promoter. We analyzed the possible binding sites of miRNA-22 in *Mtf1* promoter regions and found 12 binding sites spanning 1157 bp from -516 ~ +640 bp region in *Mtf1* promoter. Our data show that miRNA-22 binding to the *Mtf1* promoter in the nuclei of spinal neurons was augmented in CFA-injected mice in an RNA polymerase II-dependent manner; further *in vitro* reporter and *in vivo* Western-blot assays demonstrated that blocking miRNA-22 reduced reporter activity in HEK293 cells and *Mtf1* protein levels in ipsilateral spinal cord of CFA-injected mice. Conversely, upregulating miRNA-22 increased reporter activity and *Mtf1* protein levels in naïve mice. These results suggest that nuclear miRNA-22 positively regulates *Mtf1* expression in an RNA polymerase II-dependent manner. Our findings provide the first evidence that miRNA contributes in a positive manner to gene transcription in the dorsal horn under pain conditions. Interestingly, miRNA-30 can repress gene transcription by inhibiting binding of the transcription factor TFEB to the gene promoter [49]. Whether miRNA-22 also recruits transcription factors to increase *Mtf1* expression during inflammatory pain remains to be investigated.

*Mtf1*, a Zn^2+^-dependent transcription factor, is highly conserved in mammals, including human, mouse, and rat. It contains six zinc-finger motifs, three transactivation domains, and one cysteine-rich cluster, and this pseudo-tetrahedral geometry facilitates the binding with Zn^2+^ [50]. Recent limited evidence confirms the involvement of *Mtf1* in nervous system diseases, including pain. Although *Mtf1* is not required for neural differentiation after neural grafting in mice [51], it is involved in regulatory functions in various nervous tissues, such as the cortex, hippocampus, and motor neurons, via stabilizing Zn^2+^ metabolism [52] [53]. The concentration of Zn^2+^ markedly affects the threshold for visceral pain [54], suggesting that *Mtf1* may be associated with visceral nociception. In particular, deletion of *Mtf1* upregulates KLF4 expression in the ERK1/2 and AKT pathways [55], and Zn^2+^ is closely linked to activation of Cav3.2 T-channels, which shapes excitability in spinal neurons, as evidenced by the phosphorylation of ERK in the spinal cord [54]. Therefore, *Mtf1* likely regulates inflammatory pain by modulating sensitization within the spinal cord. This may explain why we detected p-ERK1/2 activity in mice with inflammatory pain. Central sensitization is caused by increased activity in excitatory spinal neurons (and/or decreased activity in inhibitory spinal neurons) and enhanced astrocytic activity. Therefore, in this study, we examined whether miRNA-22 is involved in regulating the expression of p-ERK1/2 and GFAP, which represent neuronal and astrocytic activity, respectively [56, 57]. Our results show that ERK1/2 was activated in CFA-injected mice and that inhibiting *Mtf1* abolished this activity, indicating that miRNA-22 regulates ERK1/2 activity via effects on *Mtf1*. However, it remains unclear whether miRNA-22 regulates GFAP directly or indirectly, and how *Mtf1* regulates ERK1/2 activity through intracellular Zn^2+^–Cav3.2 interactions. These underlying mechanisms need to be examined in future studies. Moreover, central sensitization can be characterized as an increase in the excitability of dorsal horn excitatory neurons or a decrease in the excitability of dorsal horn inhibitory neurons. The specific subtypes of dorsal horn neurons that participate in the antinociception mediated by knockdown of miRNA-22 remain to be determined.

Taken together, our results demonstrate that analgesic effects are achieved by inhibiting the expression of miRNA-22 in the dorsal horn of spinal cord, and that miRNA-22 regulates *Mtf1* to, in turn, influence the ERK1/2 signaling pathway. Our results provide insights into the mechanisms underlying inflammatory pain. miRNA-22 appears to be a novel mediator of inflammatory pain in the dorsal horn and may be a promising potential therapeutic target for inflammatory pain.

## Data Availability

All data supporting the findings of this study can be available from the corresponding authors upon reasonable request.

## Supplementary Materials

Supplementary 1 The descriptions of supplementary Table 1. The locomotor functions including grasping, placing and righting reflex were measured on the corresponding days after the intrathecal injection of manipulation tools. Data are mean (SEM). n =5/group; five trials. No significance; one-way ANOVA (response time vs treated groups) followed by post hoc Tukey test.

Supplementary 2 Supplementary Figure 1a-1b: miRNA-22 overexpression by intrathecal injection of Lenti-22 for 2 consecutive days increased the sensitivity to thermal (a) and mechanical (b) stimuli in week 5 after injection. n =6. ∗p <0.05, versus Vec. Data were analyzed with a using Student's t test.
