# Supplementary material for: miRNA-22 Upregulates Mtf1 in Dorsal Horn Neurons and Is Essential for Inflammatory Pain
Source: Oxid Med Cell Longev. 2022 Feb 10;2022:8622388. doi: 10.1155/2022/8622388 (PMC8886789; doi:10.1155/2022/8622388)
Supplement: Supplementary 1 — The descriptions of supplementary Table 1. The locomotor functions including grasping, placing and righting reflex were measured on the corresponding days after the intrathecal injection of manipulation tools. Data are mean (SEM). n =5/group; five trials. No significance; one-way ANOVA (response time vs treated groups) followed by post hoc Tukey test. [file 8622388.f1.pdf]

Table 1. Mean changes in locomotor function.

Treatment groups	Placing	Grasping	Righting
Sal + Scr	5(0)	5(0)	5(0)
Sal + 22-Ih	5(0)	5(0)	5(0)
CFA + Scr	5(0)	5(0)	5(0)
CFA + 22-Ih	5(0)	5(0)	5(0)
Sal + Vector	5(0)	5(0)	5(0)
Sal + LV-22	5(0)	5(0)	5(0)
CFA + Vector	5(0)	5(0)	5(0)
CFA + LV-22	5(0)	5(0)	5(0)
Scr	5(0)	5(0)	5(0)
22-mics	5(0)	5(0)	5(0)
Vector	5(0)	5(0)	5(0)
Lenti-22	5(0)	5(0)	5(0)
Sal + si-*Mtf1*	5(0)	5(0)	5(0)
CFA + si-*Mtf1*	5(0)	5(0)	5(0)
Sal + *Mtf1*-shRNA	5(0)	5(0)	5(0)
CFA + *Mtf1*-shRNA	5(0)	5(0)	5(0)
22-mics + si-*Mtf1*	5(0)	5(0)	5(0)
Lenti-22 + *Mtf1*-shRNA	5(0)	5(0)	5(0)
