# Supplementary figures and images for: miRNA-22 Upregulates Mtf1 in Dorsal Horn Neurons and Is Essential for Inflammatory Pain
Source: Oxid Med Cell Longev. 2022 Feb 10;2022:8622388. doi: 10.1155/2022/8622388 (PMC8886789; doi:10.1155/2022/8622388)

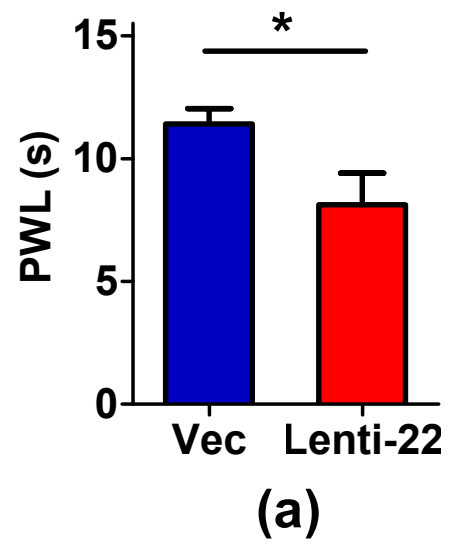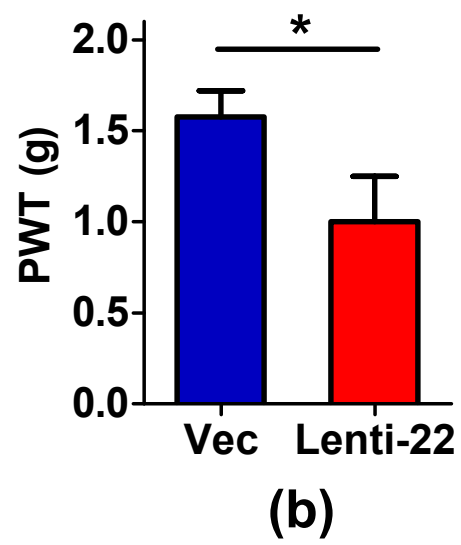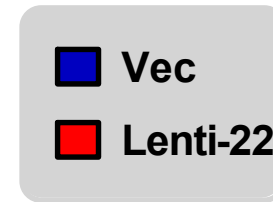

Supplement: Supplementary 2 — Supplementary Figure 1a-1b: miRNA-22 overexpression by intrathecal injection of Lenti-22 for 2 consecutive days increased the sensitivity to thermal (a) and mechanical (b) stimuli in week 5 after injection. n =6. ∗p <0.05, versus Vec. Data were analyzed with a using Student's t test. [file 8622388.f2.pdf]
